# Very Late Antigen-1 Marks Functional Tumor-Resident CD8 T Cells and Correlates with Survival of Melanoma Patients

**DOI:** 10.3389/fimmu.2016.00573

**Published:** 2016-12-12

**Authors:** Timothy Murray, Silvia A. Fuertes Marraco, Petra Baumgaertner, Natacha Bordry, Laurène Cagnon, Alena Donda, Pedro Romero, Grégory Verdeil, Daniel E. Speiser

**Affiliations:** ^1^Ludwig Cancer Research, Department of Oncology, University of Lausanne, Lausanne, Switzerland

**Keywords:** VLA-1, CD103, tissue-resident memory T cells, cancer vaccines, melanoma

## Abstract

A major limiting factor in the success of immunotherapy is tumor infiltration by CD8^+^ T cells, a process that remains poorly understood. In the present study, we characterized homing receptors expressed by human melanoma-specific CD8^+^ T cells. Our data reveal that P-selectin binding and expression of the retention integrin, very late antigen (VLA)-1, by vaccine-induced T cells correlate with longer patient survival. Furthermore, we demonstrate that CD8^+^VLA-1^+^ tumor-infiltrating lymphocytes (TILs) are highly enriched in melanoma metastases in diverse tissues. VLA-1-expressing TIL frequently co-express CD69 and CD103, indicating tissue-resident memory T cells (T_RM_) differentiation. We employed a mouse model of melanoma to further characterize VLA-1-expressing TIL. Our data show that VLA-1^+^ T_RM_ develop in murine tumors within 2 weeks, where they exhibit increased activation status, as well as superior effector functions. In addition, *in vivo* blockade of either VLA-1 or CD103 significantly impaired control of subcutaneous tumors. Together, our data indicate that VLA-1^+^ T_RM_ develop in tumors and play an important role in tumor immunity, presenting novel targets for the optimization of cancer immunotherapy.

## Introduction

CD8^+^ T cells have the capacity to recognize and kill tumor cells. The intensive development of T cell-based cancer immunotherapies over the past two decades has demonstrated remarkable therapeutic potential in both animal models and humans (1–6). However, complete tumor regression is seen in only a minority of patients, highlighting the need for better understanding and optimization of immunotherapeutic tools.

There is significant evidence that endogenous antitumor T cell responses improve clinical outcome, provided that T cells home efficiently to tumor and are retained there (7). However, numerous groups have reported discrepancies between the chemoattractants present within solid tumors and the corresponding homing receptors expressed by tumor-specific T cells (8–12). Importantly, these discrepancies can be overcome by engineering homing receptor expression in tumor-specific T cell populations (9, 12, 13) or by delivery of chemoattractants to the tumor microenvironment (10, 14). These studies provide intriguing prospects for optimization of cancer immunotherapies in the future.

Despite the established importance of T cell homing for tumor immunity, most studies have been limited to the analysis of chemokine receptors. Such studies have implicated specific chemokine receptors, including CXCR3 and CCR4 (11, 15), as being important for tumor infiltration by T cells. However, lymphocyte homing involves numerous families of cell surface receptors, including selectins, selectin ligands, and integrins, in addition to chemokine receptors. Further work is urgently needed to better characterize the role of these players in tumor infiltration by T cells.

Tissue-resident memory T cells (T_RM_) have recently been identified as a unique T cell subset that resides within non-lymphoid tissues in murine models of virus infection and are typically identified by co-expression of the surface markers, CD69 and CD103 (16–18). These cells are long lived, have high functional capacity, and are essential for efficient pathogen control at tissue barriers (19). The role of T_RM_ in cancer, however, remains unclear. Several recent reports indicate that the presence of CD103^+^ tumor-infiltrating lymphocytes (TILs) in various tumors correlates with favorable clinical outcome (20–22).

In the present study, we sought to broadly characterize the repertoire of homing receptors expressed by melanoma-specific CD8 T cells from patients with advanced disease, in circulation as well as in metastatic lesions. We further studied possible implications of homing receptor expression for tumor-infiltrating CD8 T cells using a mouse model of melanoma.

## Materials and Methods

### Melanoma Patients

Blood and tumor tissue were obtained as part of the LUD00-018 Phase I clinical trial (Trial Number NCT00112229) at the Centre Hospitalier Universitaire Vaudois (CHUV) in Lausanne. The study was conducted according to the relevant regulatory standards, upon approval by Swissmedic (the regulatory agency of Switzerland) and the “Commission d’Ethique de la Recherche Clinique de la Faculté de Biologie et de Médecine, Université de Lausanne,” which also approved the use of specimens from healthy volunteers. Patients were enrolled upon written informed consent. The trial involved serial monthly subcutaneous vaccinations of stage III/IV melanoma patients with peptides melanoma antigen recognized by T cells (Melan-A) ± Tyrosinase, together with deoxycytidyl-deoxyguanosine (CpG) 7909 and Montanide. Eighteen patients were included in the present study. These patients had a median duration of clinical follow-up of 107 months.

### Human Cell Preparation and Flow Cytometry

Peripheral blood mononuclear cells (PMBC) were directly isolated from whole blood and stored in liquid nitrogen. Human tumor tissue was manually dissociated to yield a single cell suspension, which was cryopreserved on the same day as the surgery was performed. Frozen PBMC were thawed and incubated in RPMI with 10% FCS at 37°C overnight prior to analysis. CD8^+^ cells were enriched by negative selection with a magnetic bead system (StemCell, EasySep kit). Following CD8 enrichment, cells were first stained for 20 min at 4°C with APC-labeled Melan-A peptide-MHC tetramers (TCMetrix), followed by staining for surface markers (including CD8, CCR7, CD45RA, and homing receptors) for 20 min at room temperature. Ligands for E- and P-Selectin were detected by incubating cells with recombinant E- or P-selectin (R&D) in HBSS for 20 min at 4°C, followed by staining with FITC-labeled anti-human CD62E/P (R&D) at the same time as other surface antigens. Finally, cells were stained for 20 min at 4°C with LIVE/DEAD Aqua (Life Technologies) for discrimination of dead cells. Anti-human monoclonal antibodies were purchased from BD (CD11a, CD49a, CD49b, CXCR3), Biolegend (CCR1, CCR2, CCR4, CCR5, CCR7, CCR10, CD8, CD29, CD49d, CD103, CX3CR1), Beckman Coulter (CD14, CD16, CD45RA), or eBioscience (CXCR2, CD62L). All data were acquired on a Beckman Coulter Gallios flow cytometer, and analysis was performed using FlowJo software.

### Mouse Tumor Model

CD45.2^+^ C57BL/6 mice between 6 and 10 weeks of age were used as host mice in all experiments. B16-F10 mouse melanoma cells were electroporated with an Ova-encoding plasmid and selected based on G418 resistance (23). B16-Ova and E.G7 cells were cultured for at least 5 days in RPMI, 10% FCS, prior to subcutaneous injection of 2 × 10^5^ cells into the shaved flank skin of recipient mice. CD45.1^+^ OT-1 mice were sacrificed and lymphocytes pooled from lymph nodes and spleen before retro-orbital injection (i.v.) of 1 × 10^6^ OT-1 T cells into recipient mice. 10 μg Ova peptide (SIINFEKL) and 50 μg CpG in 100 μl PBS were injected subcutaneously on the opposite flank to the tumor in order to immunize the mice. Tumor volume was measured according to length, width, and height using Vernier calipers from day 5 following tumor engraftment and every 2–3 days thereafter. The end-point for sacrifice of tumor-bearing mice was a tumor volume of 1 cm^3^, according to the Swiss regulations.

### *In Vitro* Stimulation and Expansion of OT-1 Cells

Splenocytes from CD45.1^+^ OT-1 mice were isolated as described above. Cells were then stimulated with anti-CD3 (1 μg/ml), anti-CD28 (0.1 μg/ml), and recombinant IL-2 (20 IU/ml) for 48 h. Stimulated cells were harvested, washed, counted, and resuspended in medium containing 20 ng/ml IL-15. Cells were then expanded for a further 4 days, splitting as necessary. Immediately prior to adoptive transfer, live CD8^+^ cells were sorted on a BD FACS Aria and counted. Each mouse received 5 × 10^5^ cells in PBS i.v.

### *In Vivo* Blockade of Integrins

Function-blocking antibodies against mouse very late antigen (VLA)-1 (clone Ha31/8) and CD103 (clone M290) or isotype control antibodies were purchased in “No Azide, Low Endotoxin” format from BD (VLA-1) and Bioxcell (CD103). Two hundred fifty microgram was injected intraperitoneally (i.p.) on days 6, 11, and 16 following tumor engraftment.

### Mouse Cell Preparation and Flow Cytometry

Tumors, lymph nodes, and spleens were dissected immediately after sacrifice of tumor-bearing mice. Tumor tissue was dissociated manually prior to enzymatic digestion (Mitenyi Tumor Dissociation kit). Lymph node and spleen tissue was passed through a 100-μm filter and washed twice with PBS before analysis. Anti-mouse antibodies were provided either by the Flow Cytometry Facility of the Ludwig Cancer Research Institute or purchased from BD (CD49a, CD103, CD223) or Biolegend (IFNγ and PD1). Data were acquired on a Beckman Coulter Gallios and analysis performed with FlowJo software.

### *In Vitro* Stimulation Assay

Lymphocytes from mouse tumor tissue were enriched by Ficoll separation before stimulation assays. Cells were incubated for 5 h at 37°C in the presence of PMA (10 ng/ml) and Ionomycin (500 ng/ml). Brefeldin A (BD GolgiPlug) was added to the cultures after 1 h in order to stain for intracellular cytokines.

### Statistics and Analysis

**Figure 1:**
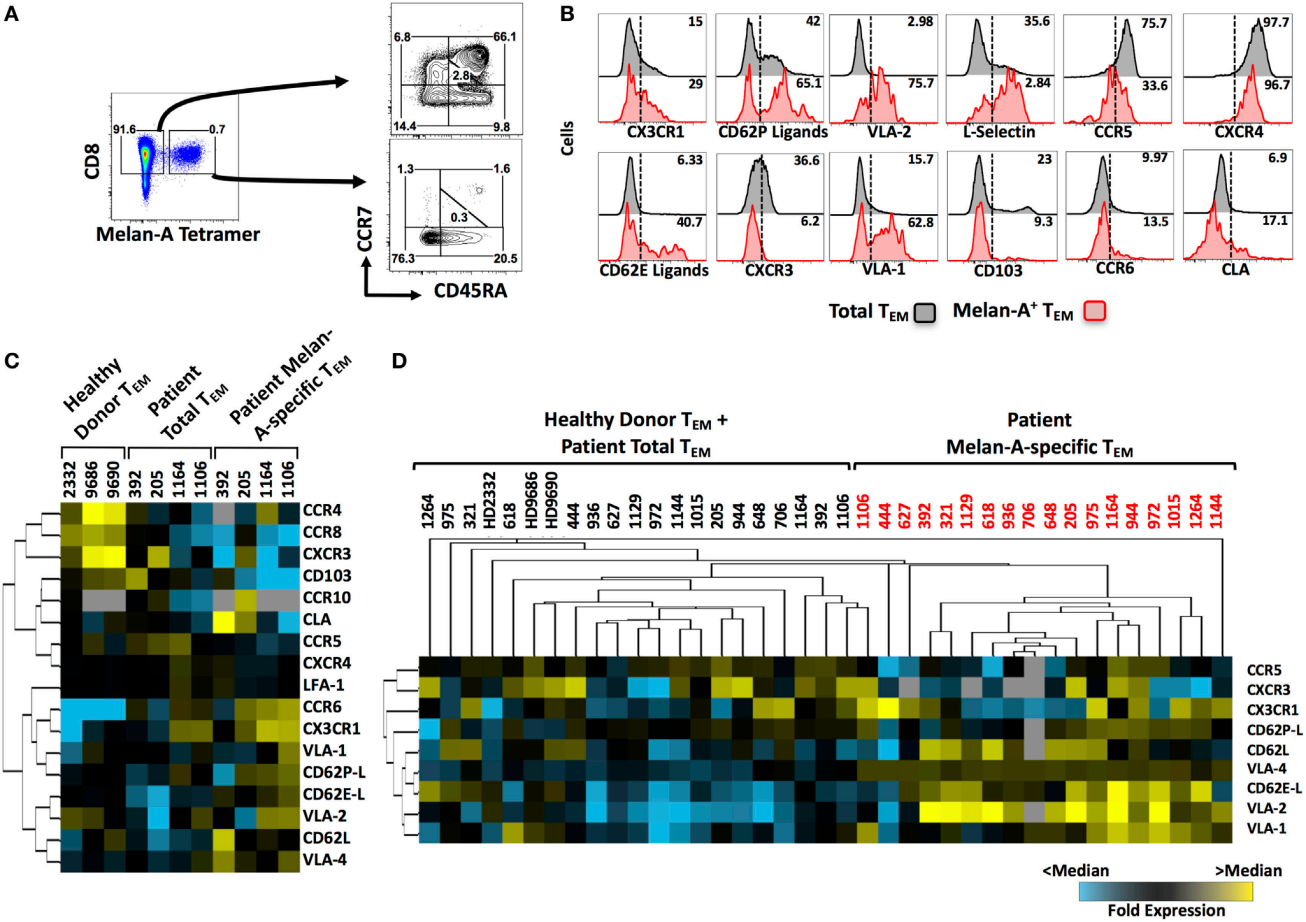
**Homing receptor expression profiling of circulating, melanoma-specific CD8^+^ T cells from peptide-vaccinated metastatic melanoma patients**. **(A)** Cryopreserved peripheral blood mononuclear cells isolated from the blood of vaccinated patients were cultured overnight and enriched for CD8^+^ cells prior to flow cytometric staining. CD8^+^ cells were stained with Melan-A tetramers, followed by fluorescently labeled antibodies against surface markers of T cell differentiation status (CCR7 and CD45RA) as well as homing receptors. **(B)** Representative histograms for several analytes on “total” or “Melan-A-specific” effector memory CD8^+^ T cells. **(C)** Heat map showing the initial screen for homing receptor expression on circulating Melan-A-specific CD8^+^ T_EM_ cells from four vaccinated patients compared to the patients’ total T_EM_ and to total T_EM_ in three healthy donors. **(D)** Heat map showing the second screen for homing receptor expression on circulating Melan-A-specific CD8^+^ T_EM_ cells from 18 vaccinated patients. Numbers on FACS plots indicate percentage positive cells within each gate **(A)** or percentage positive cells **(B)**.

Overall survival (OS) and disease-free survival (DFS) were defined as the time between enrollment in the LUD00-018 Phase I clinical trial and latest follow-up/death or relapse, respectively. Correlation between gene expression and OS was assessed by Spearman’s rank correlation coefficient, and the significance of Kaplan–Meier survival analysis was assessed by the Log-rank test, as is standard. Significance of single comparisons was assessed using the Mann–Whitney test, multiple comparisons using one-way ANOVA, and tumor growth curves using multiple unpaired *t*-tests (corrected for multiple comparisons). GraphPad Prism was used for all graphs and statistics. Tree Star software was used for hierarchical clustering of expression data (Figures 1C,D).

## Results

### Homing Receptor Expression Profiling of Circulating Melanoma-Specific CD8^+^ T Cells from Peptide-Vaccinated Metastatic Melanoma Patients

We have previously shown that subcutaneous vaccination of stage III–IV melanoma patients with the mixture of Melan-A peptide, CpG-containing oligodeoxynucleotide, and Montanide induces large numbers of circulating Melan-A-specific CD8^+^ T cells in the majority of patients treated (24). We analyzed the expression of a broad array of homing receptors (selected based on their established role in the migration of CD8 T cells from existing literature) on total and Melan-A-specific circulating CD8 T cells from these patients by flow cytometry. Melan-A-specific cells were identified by tetramer staining and, as expected, were mostly of effector memory (T_EM_) differentiation status (Figure 1A). The expression of a panel of 17 homing receptors, including selectins, selectin ligands, chemokine receptors, and integrins, was then profiled on Melan-A-specific and “total” CD8^+^ T_EM_ cells (representative examples shown in Figure 1B). The quantity of patient material was limited in some cases; therefore, we carried out an initial screen in which all homing receptors were analyzed on four patients (Figure 1C). The results of this first screen gave us an initial insight into which homing receptors were expressed by Melan-A-specific T cells. In addition, hierarchical clustering highlighted several analytes that were highly expressed by Melan-A-specific CD8^+^ T_EM_ cells compared with total T_EM_ cells from either healthy donors or patients, including: CX3CR1, CD62P-ligands, CD62E-ligands, CD62L, VLA-1, VLA-2, and VLA-4. Expression of selected analytes was subsequently investigated across a cohort of 18 patients. Unsupervised hierarchical clustering of the expression data from this second screen showed that vaccine-induced Melan-A-specific T_EM_ cells from melanoma patients display a homing receptor expression signature that is distinct from T_EM_ cells derived from the blood of healthy donors or the total T_EM_ cell pool from patient blood (Figure 1D). In addition, this second screen confirmed that certain receptors, such as CX3CR1, CD62L, several β_1_ integrins (VLA-1, VLA-2, and VLA-4), as well as ligands for E- and P-selectin, were abundantly expressed by Melan-A-specific CD8^+^ T cells relative to non-specific cells. Other analytes, such as CCR5 and CXCR3, were expressed at low levels on Melan-A-specific cells.

### P-Selectin Binding and Expression of VLA-1 by Circulating Melanoma-Specific CD8^+^ T Cells Correlate with Survival of Vaccinated Patients

**Figure 2:**
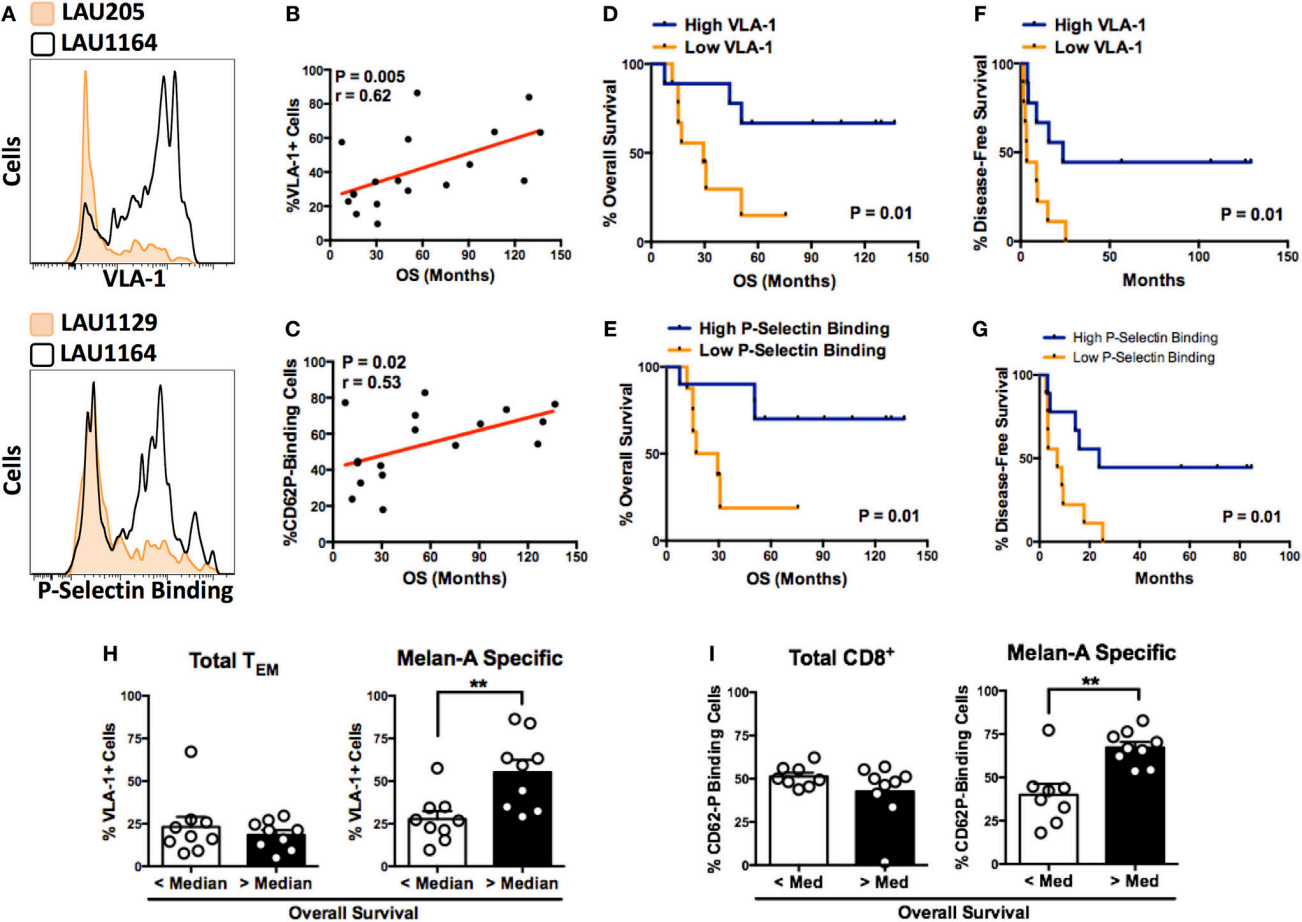
**P-selectin binding and expression of VLA-1 by circulating melanoma-specific CD8^+^ T cells correlate with survival of vaccinated patients**. **(A)** Representative histograms illustrating highly differential expression of VLA-1 (patient numbers LAU205 and LAU1164) and binding to P-selectin (patient numbers LAU1129 and LAU1164) by Melan-A-specific CD8^+^ T_EM_ cells. **(B,C)** Correlation between expression of VLA-1 **(B)** or P-selectin binding capacity **(C)** of Melan-A-specific CD8^+^ T_EM_ cells and overall patient survival. **(D,E)** Kaplan–Meier overall survival analysis of patients with Melan-A-specific effector memory CD8^+^ T cells displaying greater than (“high”) or less than (“low”) median expression of VLA-1 **(D)** or P Selectin binding **(E)**. **(F,G)** Kaplan–Meier disease-free survival analysis based on the T cell’s “high” or “low” expression of VLA-1 **(F)** or P-selectin binding **(G)**. **(H,I)** Expression of VLA-1 **(H)** or P-selectin binding **(I)** by total or Melan-A-specific CD8^+^ T_EM_ cells from patients with greater than or less than median overall survival. **(B,C)** Spearman’s rank correlation coefficient. **(D–G)** Log-rank (Mantel–Cox) test. **(H,I)** **P < 0.01 (two-tailed Mann–Whitney test).

Among those homing receptors expressed on Melan-A-specific CD8^+^ T cells, some were highly differentially expressed between individual patients (representative examples in Figure 2A). In order to assess the potential significance of differentially expressed markers, we interrogated the homing receptor expression data against two clinical parameters: OS and DFS. Interestingly, when the vaccine-induced, Melan-A-specific T cells were considered, highly significant correlations were found between OS and the expression of the retention integrin, VLA-1 (α_1_β_1_) (Figure 2B), and P-selectin (CD62P) binding (Figure 2C). We grouped patients into “high” (greater than median) or “low” (less than median) expression of VLA-1 (Figures 2D,F) or P-selectin binding (Figures 2E,G). In both cases, Kaplan–Meier survival analysis demonstrates significantly longer OS of patients in which Melan-A-specific T cells exhibited “high” expression compared with “low” expression (Figures 2D,E). The median OS of VLA-1 “high” patients was 90.8 months, in contrast to 29.4 months for VLA-1 “low” patients. Similarly, “high” P-selectin-binding patients had a longer median OS (98.8 months) as compared to patients in the “low” P-selectin-binding group (23.2 months). Statistically significant associations were also observed between both markers and prolonged DFS of the patients (Figures 2F,G). Importantly, the associations between survival and VLA-1 expression or P-selectin-binding were restricted to the Melan-A-specific pool. No differences in the expression of either analyte were observed between “total” T_EM_ cells of patients with “longer than” or “shorter than” median OS (Figures 2H,I).

### CD8^+^ T_RM_ Cells That Express VLA-1 Are Highly Enriched in Human Melanoma Metastases Compared to Blood Circulation

We, next, interrogated the homing profiles of CD8^+^ T cells within the TIL pool of metastatic melanoma lesions from the same cohort of patients. We hypothesized that receptors that contribute to tumor homing would likely be expressed by a large proportion of cells that successfully infiltrate and remain within the tumor. Thus, we investigated the expression of the same panel of homing analytes on CD8^+^ T cells derived from melanoma metastases in various organs including skin, brain, and lung.

**Figure 3:**
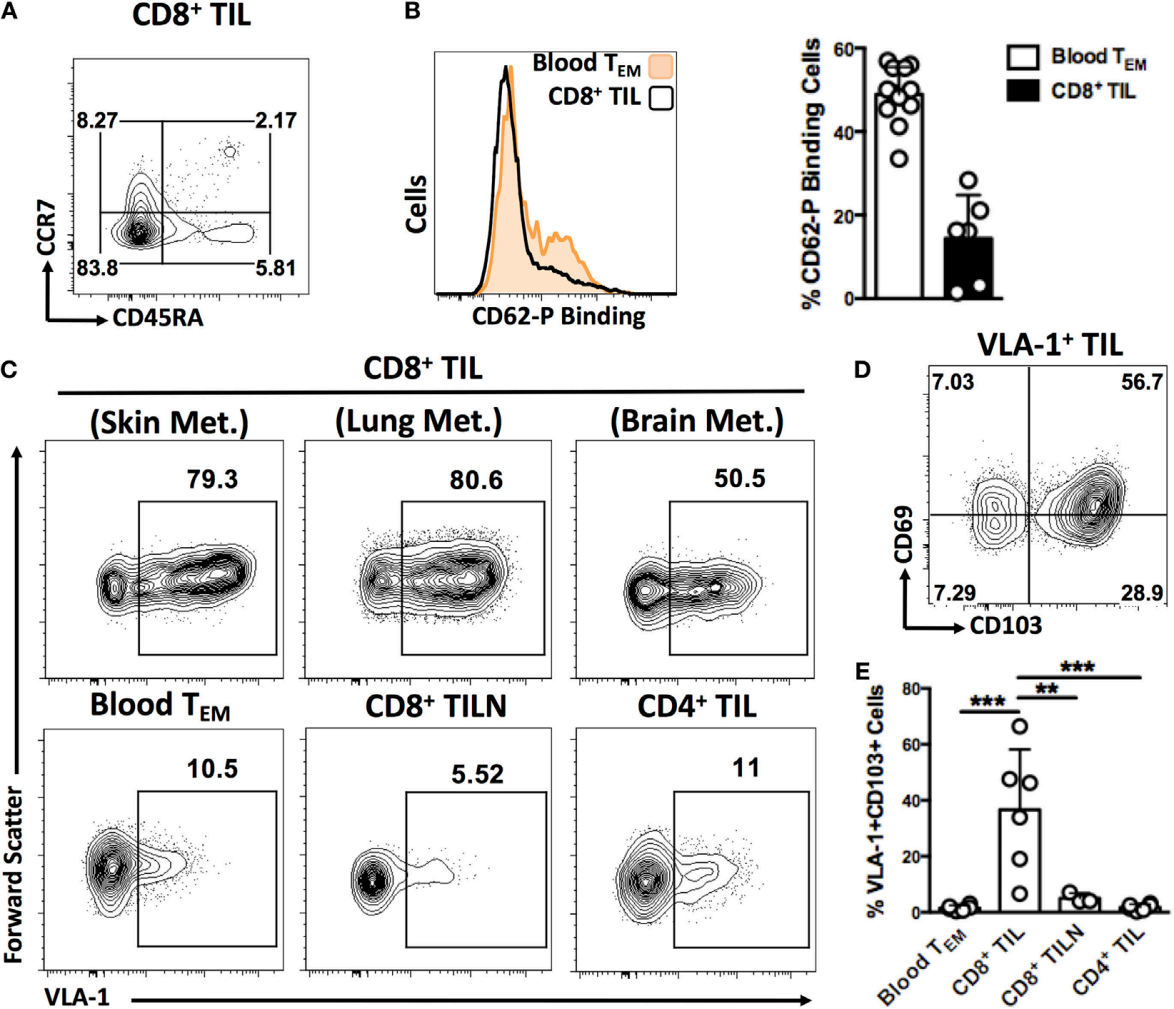
**CD8^+^ T cells expressing VLA-1 and other integrins are highly enriched in human melanoma metastases compared to blood circulation**. **(A)** Representative dot plot illustrating the predominantly T_EM_ differentiation status of the CD8^+^ TIL analyzed in this study. **(B)** Representative histograms and quantification of P-Selectin binding by CD8^+^ T_EM_ cells isolated from blood circulation and melanoma tumors. **(C)** Representative dot plots of VLA-1 and CD103 expression on CD8^+^ T_EM_ cells derived from melanoma metastases in skin, lung, and brain, blood circulation, metastatic lymph nodes (TILN), and tumor-derived CD4^+^ T cells. **(D)** Representative dot plot showing expression of T_RM_ markers, CD69 and CD103, by VLA-1-expressing TIL. **(E)** Quantification of cells co-expressing VLA-1 and CD103 in the cellular compartments indicated. Numbers on FACS plots represent percentage cells in each gate **(A,C)**. **(D)** **P < 0.01, ***P < 0.005 (One-way ANOVA, Tukey’s multiple comparisons test).

As expected, tumor-infiltrating T cells were mostly of effector memory differentiation status with very few naïve cells (Figure 3A). Certain activation markers, such as CXCR3 and CD69, were broadly expressed, whereas L-selectin expression was seen on very few cells (Figure S1 in Supplementary Material). P-selectin binding by CD8^+^ TIL was low in comparison with circulating CD8^+^ cells (Figure 3B), despite the correlation we had previously observed between P-selectin binding of circulating Melan-A-specific T cells and patient survival. This may reflect a down-modulation of P-selectin ligands following extravasation from blood. In contrast, VLA-1-expressing CD8^+^ cells were highly enriched in TIL relative to circulating cells, irrespective of the organ in which the metastasis arose (Figure 3C). We also analyzed CD8^+^ T cells derived from tumor-infiltrated lymph nodes (TILN) as well as CD4^+^ TIL and found no such enrichment for VLA-1 expression, suggesting a homing mechanism unique to CD8^+^ cells in the tumor microenvironment (Figure 3C). Interestingly, VLA-1 was frequently co-expressed with archetypal markers of T_RM_ differentiation, including CD69 and integrin CD103 (16–18) (Figure 3D). Indeed, the co-expression of both VLA-1 and CD103 was almost entirely absent from all other cellular compartments analyzed (Figure 3E). These data suggest a specialized migratory phenotype specific to CD8^+^ T cells that successfully infiltrate and remain within melanoma metastases of diverse tissue origin.

### In Subcutaneous Murine Tumors, CD8^+^ TIL Display an Integrin Expression Profile That Is Unique to the Tumor Microenvironment and Is Dependent on Subcutaneous Immunization

In order to further investigate integrin function in the context of antitumor immunity by CD8^+^ T cells, we chose to use a mouse model of melanoma for the next phase of this study. Our initial aim was to determine whether the VLA-1^+^CD103^+^ phenotype seen on CD8^+^ TIL from human melanoma was present on TIL derived from mouse melanoma. This would imply that similar mechanisms of retention and/or survival of T cells within tumors are conserved between human and mouse, and that murine models provide a relevant pre-clinical system.

**Figure 4:**
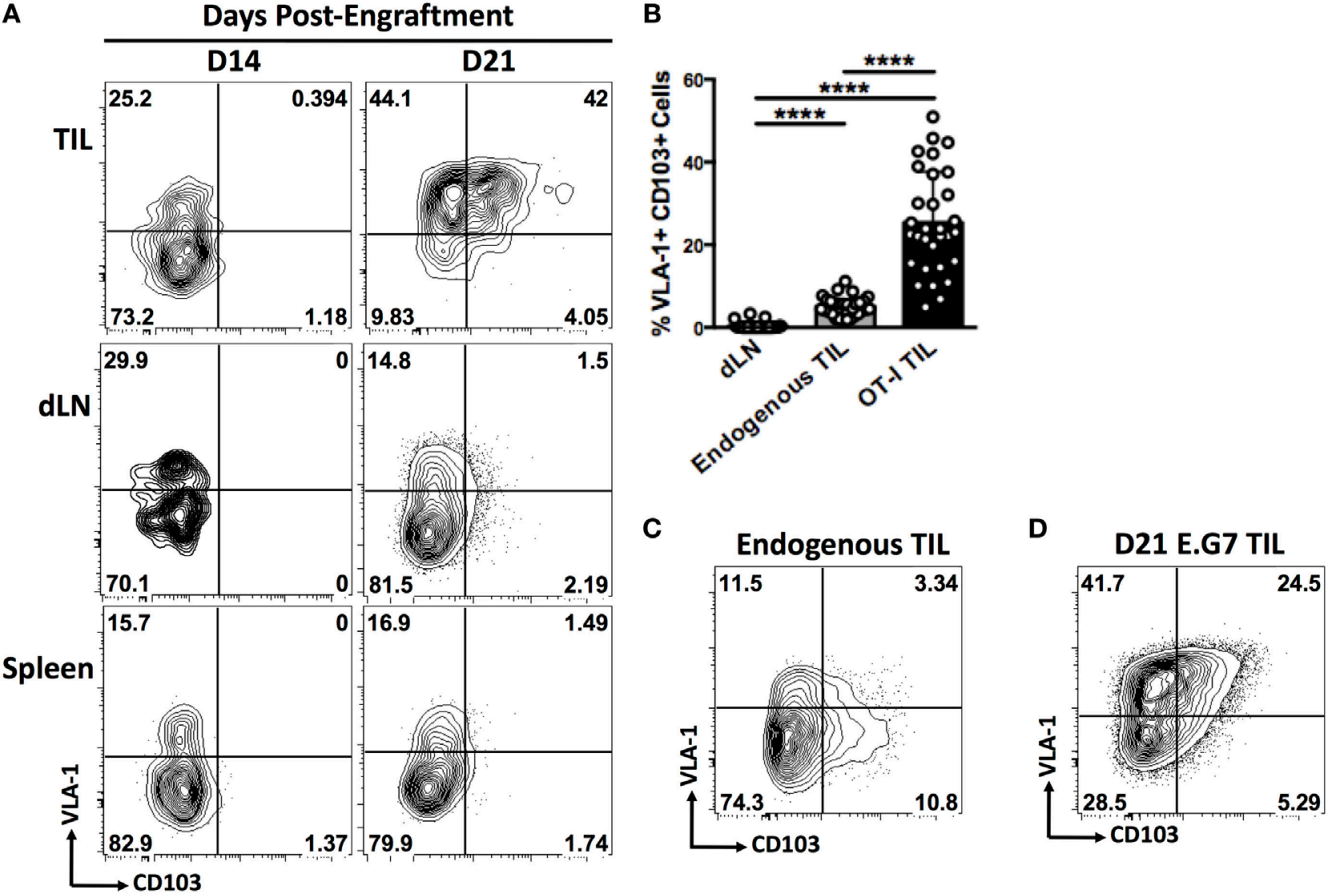
**In subcutaneous murine tumors, CD8^+^ TIL display an integrin-expression profile that is unique to the tumor microenvironment and is dependent on subcutaneous immunization**. **(A)** Representative dot plots of VLA-1 and CD103 expression by OT-1 T cells derived from tumor, tumor-draining lymph node (dLN), and spleen of mice bearing subcutaneous B16-Ova tumors on day 14 or day 21. **(B)** Quantification of co-expression of VLA-1 and CD103 by endogenous CD8^+^ T cells derived from dLN, tumor, or OT-1 T cells derived from tumor. **(C)** Representative dot plot of VLA-1 and CD103 expression by endogenous CD8^+^ T cells derived from B16-Ova tumors. **(D)** Representative dot plot showing co-expression of VLA-1 and CD103 by OT-1 T cells derived from a subcutaneous E.G7. Numbers on FACS plots indicate percentage positive cells in each gate **(A,C,D)**. **(B)** ****P < 0.0001 (one-way ANOVA, Tukey’s multiple comparisons test).

We carried out subcutaneous engraftment of Ova-expressing B16 melanoma cells in the flank of C57BL/6 mice (day 0), followed by adoptive transfer of 1 × 10^6^ OT-1 T cells on day 7, and immunization with Ova peptide and CpG on day 8. Mice were sacrificed on day 14 or day 21 and integrin expression was analyzed on CD8^+^ T cells derived from tumor, tumor-draining lymph nodes (dLN), and spleen (Figure 4A). At day 14, OT-1 TIL exhibited VLA-1 expression comparable with that seen in dLN and spleen and were negative for CD103. By day 21, however, VLA-1 expression was observed on a large majority of OT-1 TIL, and up to 50% of these cells displayed the VLA-1^+^CD103^+^ adhesion signature that we had previously observed in human melanoma metastases (Figures 4A,B). This phenotype was entirely absent from OT-1 T cells derived from either spleen or tumor-draining lymph nodes at both time points (Figures 4A,B).

We also observed expression of VLA-1 and CD103 on endogenous CD8^+^ TIL, but to a significantly lesser extent than in the OT-1 compartment (Figures 4B,C). It is likely that the proportion of endogenous TIL responding to antigen is significantly lower than in the OT-1 T cell pool, which responds to the immunodominant OVA epitope in B16-OVA tumor cells. There are good reasons to assume that the intratumoral induction of VLA-1 and CD103 expression is, in part, dependent on efficient antigen recognition within the tissue, as this process has been well described in both cases (25–27).

In order to establish whether intratumoral integrin expression by T cells is specific to the melanoma microenvironment, we engrafted mice subcutaneously with an Ova-expressing mouse lymphoma cell line, E.G7. Despite the distinct tissue origin of this tumor cell line, OT-1 TIL showed a similar integrin-expression profile to that seen in melanoma tumors (Figure 4D), suggesting a general mechanism of homing or retention within subcutaneous tumors.

Finally, we addressed the question of whether intratumoral integrin expression was dependent on the context of T cell priming. We engrafted mice with B16-Ova tumors and, rather than performing adoptive transfer of naïve OT-1 cells followed by subcutaneous immunization with CpG and SIINFEKL, we stimulated and expanded OT-1 cells *in vitro* prior to transfer into tumor-bearing mice. Analysis of OT-1 TIL from mice having received *in vitro*-stimulated cells revealed very low expression of both VLA-1 and CD103 compared with TIL from mice having been immunized with CpG and SIINFEKL peptide (Figure S2 in Supplementary Material). This data demonstrate that adoptive cell transfer of *in vitro*-expanded TIL may generate effector cells which lack the expression of homing receptors important for tumor infiltration and retention.

### VLA-1-Expressing OT-1 TIL Are Resident Memory T cells That Are Highly Activated and Exhibit Superior Effector Functions

**Figure 5:**
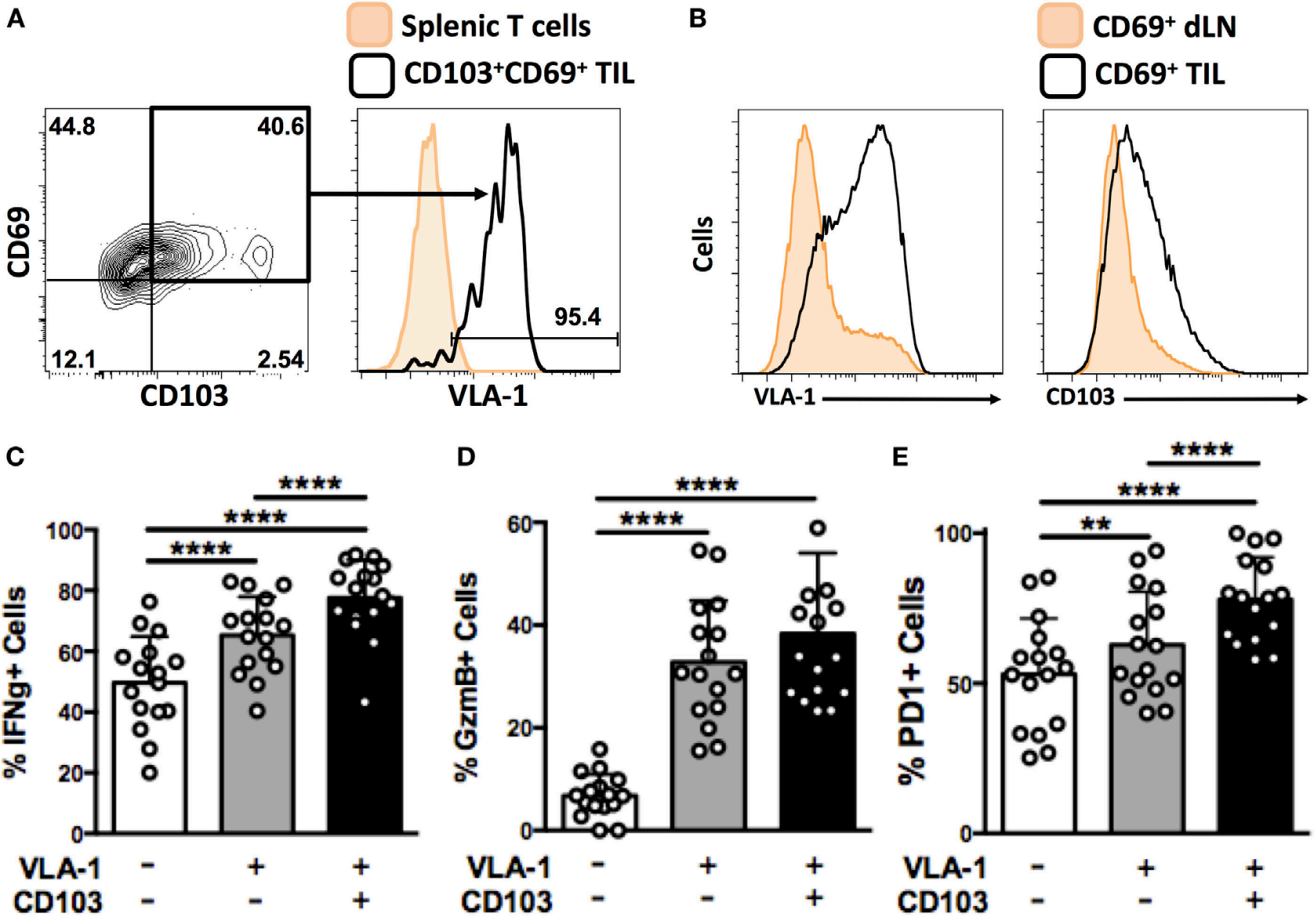
**VLA-1-expressing OT-1 TIL are resident memory cells that are highly activated and exhibit superior effector functions**. **(A)** Representative dot plot and histogram showing expression of CD69 and CD103 expression on OT-1 T cells derived from B16-Ova tumors at day 21, and VLA-1 expression by the CD69^+^CD103^+^ subset. **(B)** Expression of VLA-1 and CD103 by activated (CD69^+^) cells derived from tumor-draining lymph node or tumor. **(C–E)** Quantification of IFNγ secretion **(C)**, granzyme B expression **(D)**, and PD-1 expression **(E)** by integrin-positive or -negative OT-1 T cells derived from B16-Ova tumors upon *ex vivo* re-stimulation. Numbers on FACS plots indicate percentage of cells within each gate **(A)**. **(C–E)** **P < 0.01, ****P < 0.0001 (one-way ANOVA, Tukey’s multiple comparisons test).

Expression of CD49a, the alpha subunit of VLA-1, has previously been reported on virus-induced T_RM_ cells from murine skin (17) and brain (28). This phenotype has not previously been investigated in the context of cancer. In our system, around 90% of CD103^+^CD69^+^ OT-1 TIL derived from B16-Ova tumors at day 21 also expressed VLA-1, strongly indicating that VLA-1 is induced as part of the program of T_RM_ differentiation in tumors (Figure 5A), similar to previous reports from models of virus infection.

CD69 is a marker of activation. A small number of CD69^+^ OT-1 T cells were also consistently found in the dLN of mice. Therefore, in order to distinguish between integrin expression induced by cellular activation or tissue infiltration, we compared the integrin-expression profiles of CD69^+^ cells in tumor (TIL) versus tumor-draining lymph nodes. CD69^+^ cells from the two tissue compartments showed strikingly different expression of VLA-1 and CD103, with only a small fraction of CD69^+^ OT-1 in dLN staining positively (Figure 5B) compared to the generalized expression of VLA-1 and CD103 in TIL. Together, these data support the notion that VLA-1 and CD103 expression on CD8^+^ TIL represents a tumor-specific adhesion phenotype, rather than simply an extravasated or activation-induced response.

To further investigate the functionality of integrin-expressing CD8^+^ TIL, we isolated OT-1 T cells from B16 tumor digests and performed *in vitro* re-stimulation assays. Cytokine secretion and degranulation of VLA-1- and CD103-expressing TIL was analyzed in comparison to integrin-negative TIL. We observed that a significantly higher proportion of OT-1 TIL expressing VLA-1 or co-expressing both VLA-1 and CD103 secreted IFNγ upon re-stimulation, as compared to their integrin-negative counterparts (Figure 5C). The same was true for granzyme B expression (Figure 5D). Finally, we quantified expression of the activation marker, PD-1 (29), on integrin-expressing OT-1 TIL. Again, we observed that OT-1 TIL positive for VLA-1, or co-expressing both VLA-1 and CD103, were significantly more activated than integrin-negative OT-1 (Figure 5E). Together, these data indicate that expression of VLA-1 on tumor-derived T_RM_ cells correlates with a higher degree of activation and functionality, superior to that of cells that do not exhibit this retention phenotype.

### *In Vivo* Blockade of VLA-1 and CD103 Impairs Control of Subcutaneous B16-Ova Tumors

**Figure 6:**
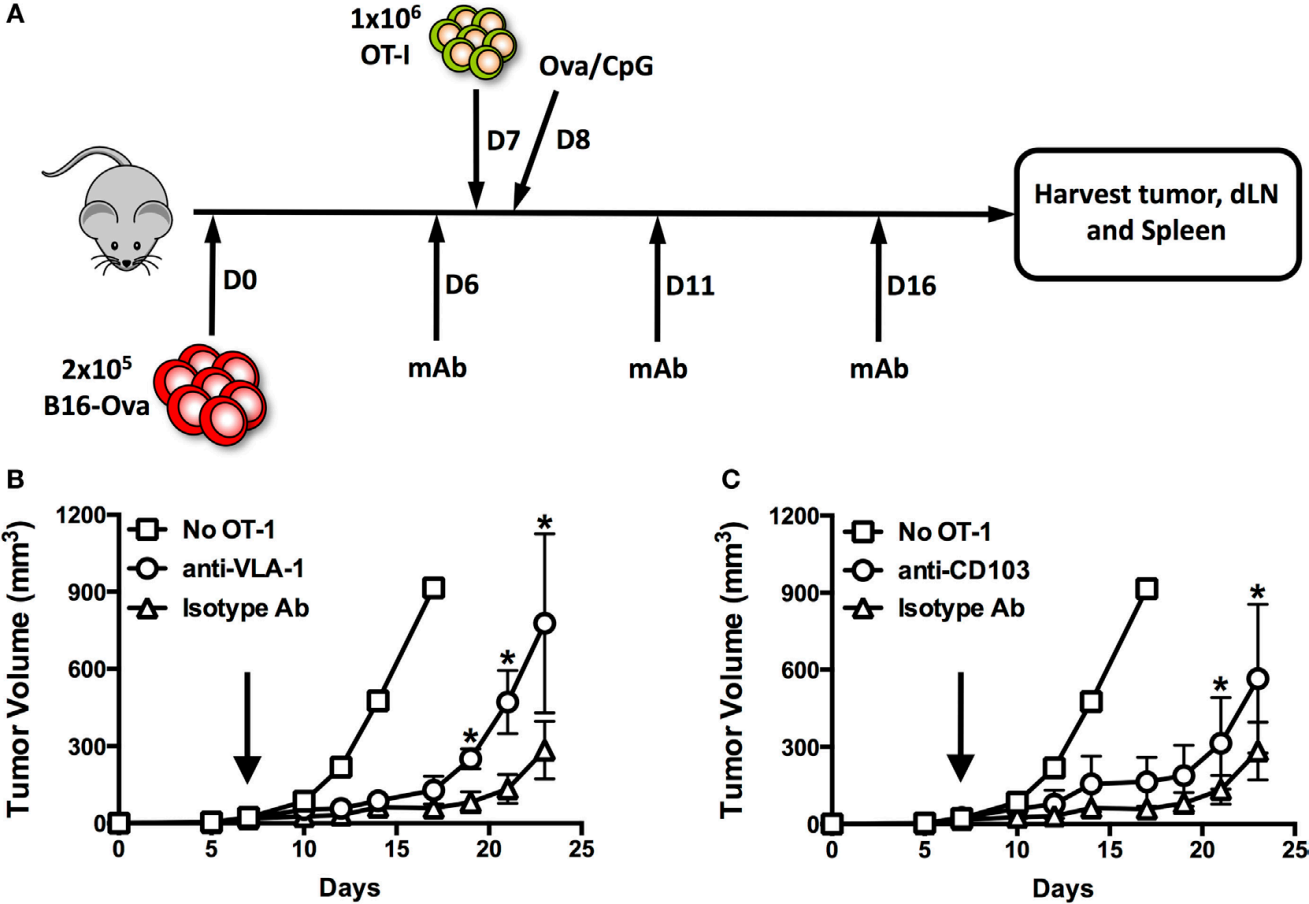
***In vivo* blockade of VLA-1 and CD103 impairs control of subcutaneous B16-Ova tumors**. **(A)** Scheme illustrating engraftment, adoptive transfer of OT-1, and systemic treatment with integrin function-blocking monoclonal antibodies. **(B,C)** Growth of subcutaneous B16-Ova tumors in mice receiving anti-VLA-1 **(B)** or anti-CD103 **(C)** blocking antibodies or isotype control antibody injections i.p. Black arrows indicate adoptive transfer of OT-1 T cells on day 7. *P < 0.05, **P < 0.01 (multiple unpaired *t*-tests, corrected for multiple comparisons, Holm–Sidak method).

In order to assess the importance of integrin expression for tumor infiltration and/or functionality of CD8^+^ T cells, we treated mice bearing subcutaneous B16 tumors with function-blocking monoclonal antibodies against VLA-1 and CD103. Antibodies and OT-1 T cells were administered as indicated in Figure 6A. While control mice (treated with isotype antibodies) showed adequately delayed tumor growth, we observed significantly impaired tumor control in mice treated with either anti-VLA-1 (Figure 6B) or anti-CD103 (Figure 6C) antibodies. This impairment was more pronounced when VLA-1 was blocked. These results clearly indicate that integrin blockade targeting VLA-1 or CD103 has a negative impact on antitumor immunity.

## Discussion

In the present study, we first sought to characterize homing receptor expression by circulating melanoma-specific CD8^+^ T cells in humans. In doing so, we considered a much broader array of analytes than has been investigated in several previous studies (8, 10, 15). We found that two of the parameters analyzed, VLA-1 expression, and P-selectin binding, correlated significantly with overall and DFS in a cohort of advanced melanoma patients, suggesting important roles in tumor homing. We then investigated the expression of those analytes on CD8^+^ T cells derived from metastatic lesions from various organs including skin, lung, and brain. While we did not observe significant expression of P-selectin ligands by CD8^+^ TIL, we saw specific enrichment for cells expressing VLA-1, consistent with previous reports in melanoma bowel metastases (30) and head and neck tumors (31). Moreover, co-expression of VLA-1 together with CD69 and CD103, the archetypal markers of T_RM_ differentiation, was observed on a large proportion of cells that infiltrate tumors. To our knowledge, this is the first indication that T_RM_ develop in tumors and display a VLA-1^+^CD69^+^CD103^+^ retention phenotype.

Both VLA-1 and CD103 are so-called “retention” integrins, proposed to underlie the long-term retention of T cells within peripheral tissues (16–18). They bind to distinct ligands: extracellular matrix collagen in the case of VLA-1 (32) and epithelial E-cadherin in the case of CD103 (33). Moreover, there is evidence that expression of retention integrins correlates with superior T cell effector functions and long-term cell survival (34–36). Therefore, we chose to further investigate the role of these molecules in the antitumor immune response by means of a mouse tumor model.

We observed that a large proportion of adoptively transferred OT-1 T cells derived from B16-Ova tumors also express VLA-1, frequently in combination with CD103, suggesting similar mechanisms of T cell adhesion within human and mouse tumors. Interestingly, this phenotype appeared 2 weeks post-transfer (but not earlier at 1 week). Furthermore, retention integrins were largely co-expressed with CD69, suggesting T_RM_ differentiation status according to existing literature (16–18). Studies in murine models of viral infection have demonstrated that significant proportions of T_RM_ cells develop 2–3 weeks after infection (17), consistent with the time frame in which integrin^+^CD69^+^ cells developed within subcutaneous tumors in our model. CD8^+^ T cells that co-expressed VLA-1 and CD103 were virtually absent from tumor-draining lymph nodes and spleen, as well as the CD4^+^ T cell compartment, thus providing further evidence that CD8+ T_RM_-like cells develop specifically within tumors, an intriguing concept that has not been investigated to date. Since these are transgenic T cell receptor (TCR) T cells, the origin of antigen-specific T_RM_-like TILs is unambiguous in our system, as they are derived from naïve OT-1 T cells that were adoptively transferred into tumor-bearing mice.

Interestingly, similar T_RM_-like phenotypes were observed on OT-1 TIL regardless of whether host mice were engrafted with Ova-expressing B16 melanoma or E.G7 lymphoma cell lines, indicating that this mechanism of T cell adhesion within the tumor occurs beyond the context of melanoma. This finding is, however, in contrast to a recent report showing that antigen-specific T cells infiltrating subcutaneous non-small-cell lung tumors do not express VLA-1 (31). This discrepancy may be attributable to the route of vaccination used (intranasal versus subcutaneous) or the time point at which TIL were harvested (10 days versus 14 days post-vaccination). Alternatively, secretion of cytokines required for T_RM_ formation, such as TGFβ and IL-15 (37), may differ in the microenvironment of lung tumors compared with melanoma and lymphoma. Our finding that T_RM_ cells fail to develop within the tumors of mice receiving *in vitro*-expanded T cells may have important implications for adoptive cell transfer in the clinic. Specifically, considering that T_RM_ formation is important for T cell-mediated antitumor immunity, as indicated by our findings together with recent reports in ovarian (20), lung (21), and bladder (22) cancer, it is noteworthy that adoptive cell transfer protocols (that require T cell expansion *in vitro*) may generate effector T cells with non-optimal homing receptor expression.

Finally, we observed that OT-1 TIL expressing VLA-1 and CD103 were more activated and displayed superior effector functions to their integrin-negative counterparts. Moreover, treatment of tumor-bearing mice with function-blocking monoclonal antibodies against either VLA-1 or CD103 *in vivo* led to significantly faster tumor growth compared to isotype control-treated mice.

To date, T_RM_ cells have been described as a distinct subset of virus-specific memory T cells in non-lymphoid tissues (16, 38, 39), with a unique transcriptional profile (17). These cells are known to be long lived and have high functional potential, to readily secrete inflammatory cytokines and chemokines upon antigen rechallenge (40), and even to induce a state of “pathogen alert” by diverse cells in the surrounding tissue (19). In our study, we clearly demonstrate that T_RM_ cells specifically develop within tumors (in human and mouse) in addition to models of viral infection, in which the current paradigm was established.

Intriguingly, VLA-1- and CD103-mediated retention of T cells depends on TGFβ (TGFβ induces VLA-1 and CD103 expression *in situ*) (17, 18, 41), while this cytokine is commonly described to suppress T cell immunity, particularly in the tumor microenvironment (42). In fact, it is becoming increasingly clear that the effects of TGFβ on CD8 T cells are dependent on the differentiation subset in question, as well as the tissue and temporal context. While TGFβ appears to regulate the proliferation and survival of effector T cells during the early stages of the immune response (43–45), prolonged exposure to TGFβ within non-lymphoid tissues is required for proper development of T_RM_ populations that provide front-line defense upon rechallenge (18, 44). These findings, in the context of the data we present here, are of significant importance for cancer immunology. Future research must focus on detailed characterization of the various subsets and differentiation states of intratumoral T cells, with the aim to understand their regulation and interdependence.

Altogether, our findings reveal that P-selectin binding and expression of integrin VLA-1 by circulating Melan-A specific CD8^+^ T cells correlate significantly with the survival of melanoma patients, suggesting an important role in antitumor immunity and the potential to use of these analytes as biomarkers. Moreover, we demonstrate that VLA-1 expressing CD8^+^ T cells are highly enriched in melanoma metastases in various tissues, consistent with other recent reports in melanoma (30) and mucosal tumors in lung and head/neck (31). Within human melanoma tumors, VLA-1 was frequently expressed in combination with both CD69 and CD103, the archetypal markers for T_RM_ differentiation, as has been reported on virus-specific T_RM_ from murine skin (17) and brain (28). We show that this phenotype is unique to the CD8^+^ TIL compartment.

Three recent reports have demonstrated that the presence of CD8^+^CD103^+^ TIL is associated with more favorable prognosis in ovarian (46), lung (21), and bladder cancer (22), but the importance of T_RM_ cells for antitumor immunity remains to be determined. We now show that T_RM_ cells expressing both VLA-1 and CD103 develop in subcutaneous murine melanoma and lymphoma tumors within 2 weeks. These cells are highly activated and exhibit superior effector functions to their integrin-negative counterparts. Importantly, *in vivo* blockade of either VLA-1 or CD103 impairs the control of subcutaneously engrafted melanoma tumors. These data provide the first insight into the critical formation and functionality of T_RM_ cells within tumors, which is of significant importance for the optimization of T cell-based cancer immunotherapy.

## Author Contributions

Conception and design: TM, SM, GV, and DS. Acquisition of data (provided animals, managed patients, provided facilities): TM, SM, PB, NB, LC, AD, PR, GV, and DS. Analysis and interpretation of data: TM, SM, GV, and DS. Writing, review, and/or revision of the manuscript: TM, GV, SM, and DS. Study supervision: DS.

## Conflict of Interest Statement

The authors declare that the research was conducted in the absence of any commercial or financial relationships that could be construed as a potential conflict of interest.

## Supplementary Material

Figure S1 **Activation status of CD8^+^ T cells from melanoma metastases**. Representative flow cytometry plots showing the gating of lymphocytes of tumor-derived effector memory T cells from resected metastatic melanoma. Histograms show expression of CXCR3, CD69, and CD62L on tumor-derived T cells, in comparison with blood-derived cells of equivalent differentiation status.

Figure S2 **Expression of retention integrins, VLA-1, and CD103, by *in vitro* stimulated/expanded, adoptively transferred OT-1 T cells derived from B16-Ova tumors of mice having been subcutaneously vaccinated or not**.

## Abbreviations

TIL, tumor-infiltrating lymphocytes; TILN, tumor-infiltrated lymph nodes; dLN, tumor-draining lymph node; TEM, effector memory T cell; TRM, tissue-resident memory T cells; CpG, deoxycytidyl-deoxyguanosine; Melan-A (Melan-A/MART-1), melanoma antigen recognized by T cells; TCR, T cell receptor.
