# Supplementary figures and images for: Very Late Antigen-1 Marks Functional Tumor-Resident CD8 T Cells and Correlates with Survival of Melanoma Patients
Source: Front Immunol. 2016 Dec 12;7:573. doi: 10.3389/fimmu.2016.00573 (PMC5150229; doi:10.3389/fimmu.2016.00573)

# Supplementary Fig 1

■ Blood CD8<sup>+</sup> T<sub>EM</sub>  
□ CD8<sup>+</sup> TIL

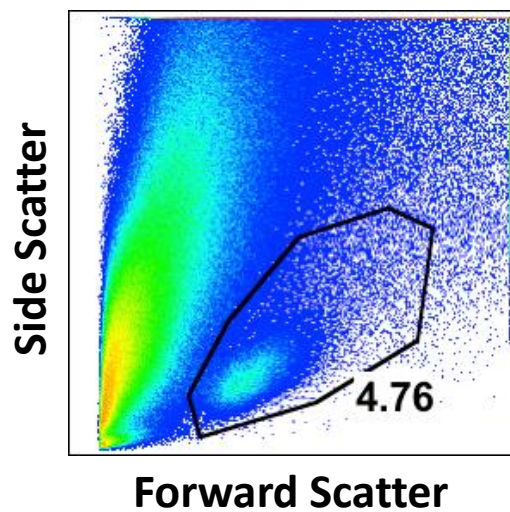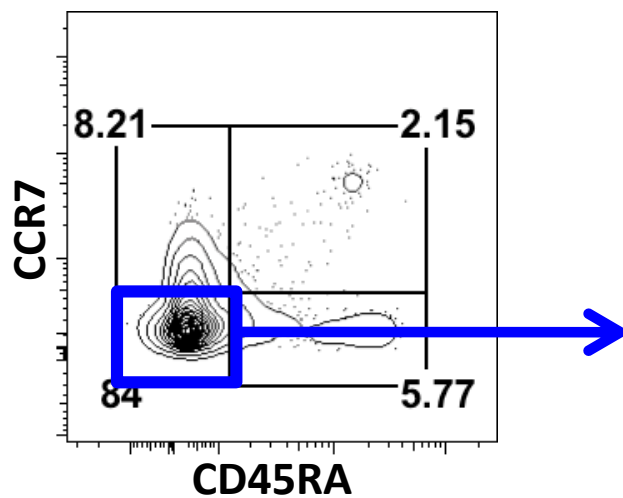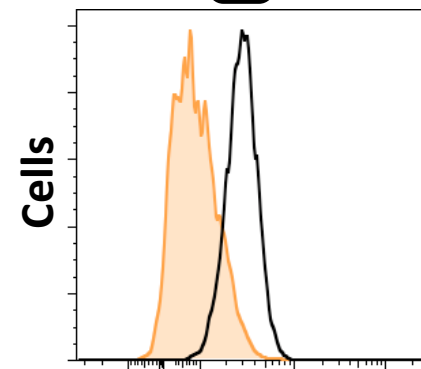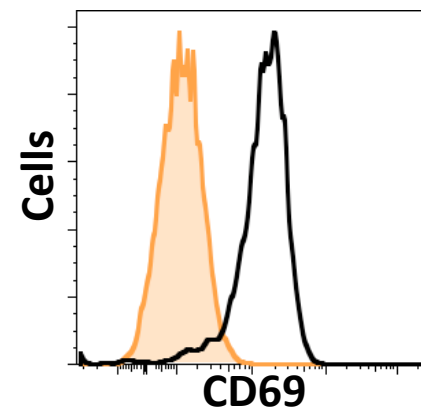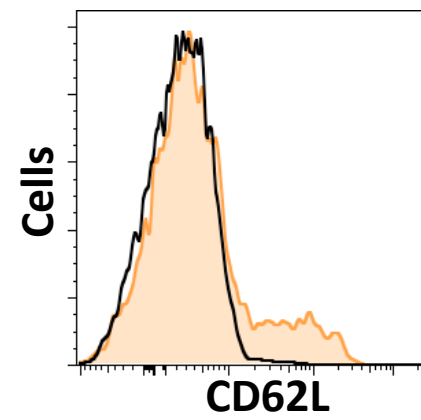

Supplement: Figure S1 — Activation status of CD8+ T cells from melanoma metastases. Representative flow cytometry plots showing the gating of lymphocytes of tumor-derived effector memory T cells from resected metastatic melanoma. Histograms show expression of CXCR3, CD69, and CD62L on tumor-derived T cells, in comparison with blood-derived cells of equivalent differentiation status. [file Image_1.PDF]

# Supplementary Fig 2

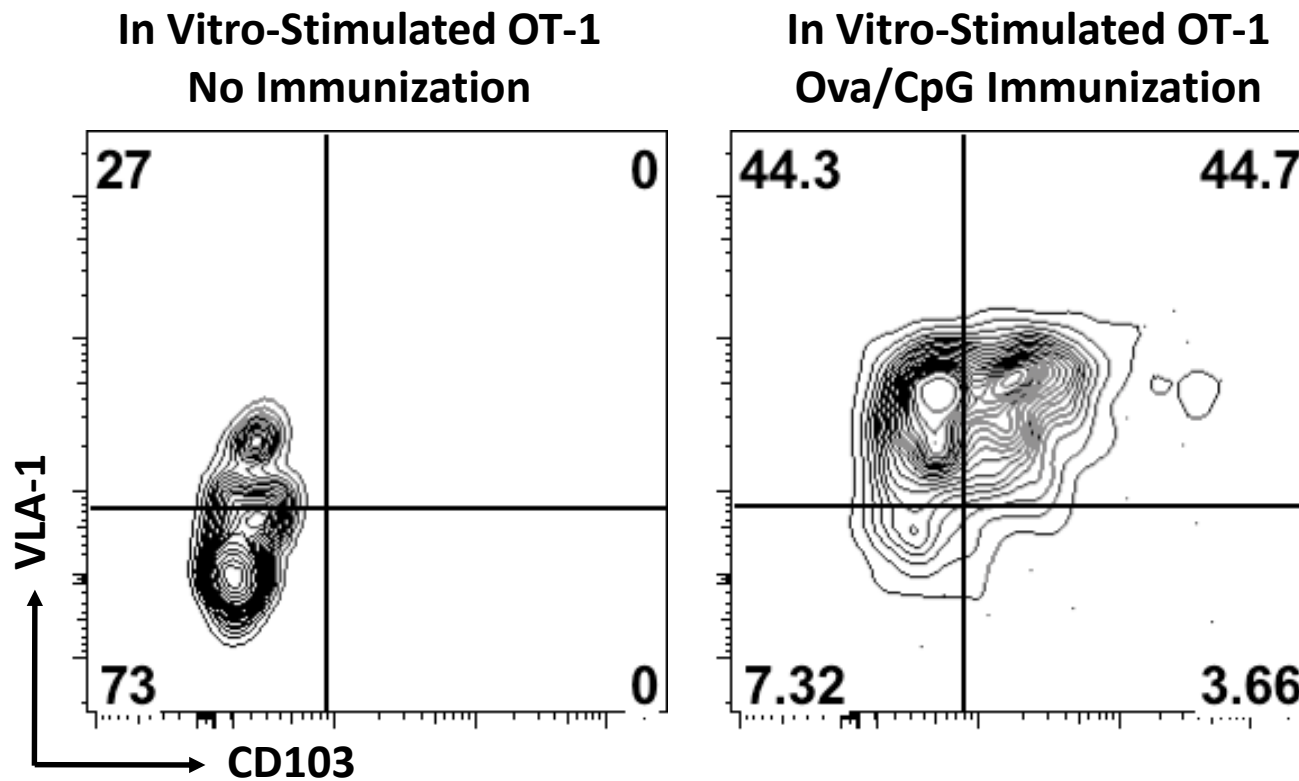

Supplement: Figure S2 — Expression of retention integrins, VLA-1, and CD103, by in vitro stimulated/expanded, adoptively transferred OT-1 T cells derived from B16-Ova tumors of mice having been subcutaneously vaccinated or not. [file Image_2.PDF]
